# Functional inference of long non-coding RNAs through exploration of highly conserved regions

**DOI:** 10.3389/fgene.2023.1177259

**Published:** 2023-05-16

**Authors:** Zhongpeng Liu, Tianbin Guo, Zhuoda Yin, Yanluo Zeng, Haiwen Liu, Hongyan Yin

**Affiliations:** ^1^ Hainan Key Laboratory for Sustainable Utilization of Tropical Bioresources, College of Tropical Crops, Hainan University, Haikou, China; ^2^ TJ-YZ School of Network Science, Haikou University of Economics, Haikou, China

**Keywords:** homolog clustering, HCR, *XACT*, *LINC00461*, functional inference

## Abstract

**Background:** Long non-coding RNAs (lncRNAs), which are generally less functionally characterized or less annotated, evolve more rapidly than mRNAs and substantially possess fewer sequence conservation patterns than protein-coding genes across divergent species. People assume that the functional inference could be conducted on the evolutionarily conserved long non-coding RNAs as they are most likely to be functional. In the past decades, substantial progress has been made in discussions on the evolutionary conservation of non-coding genomic regions from multiple perspectives. However, understanding their conservation and the functions associated with sequence conservation in relation to further corresponding phenotypic variability or disorders still remains incomplete.

**Results:** Accordingly, we determined a highly conserved region (HCR) to verify the sequence conservation among long non-coding RNAs and systematically profiled homologous long non-coding RNA clusters in humans and mice based on the detection of highly conserved regions. Moreover, according to homolog clustering, we explored the potential function inference via highly conserved regions on representative long non-coding RNAs. On lncRNA *XACT,* we investigated the potential functional competence between *XACT* and lncRNA *XIST* by recruiting miRNA-29a, regulating the downstream target genes. In addition, on lncRNA *LINC00461,* we examined the interaction relationship between *LINC00461* and SND1. This interaction or association may be perturbed during the progression of glioma. In addition, we have constructed a website with user-friendly web interfaces for searching, analyzing, and downloading to present the homologous clusters of humans and mice.

**Conclusion:** Collectively, homolog clustering via the highly conserved region definition and detection on long non-coding RNAs, as well as the functional explorations on representative sequences in our research, would provide new evidence for the potential function of long non-coding RNAs. Our results on the remarkable roles of long non-coding RNAs would presumably provide a new theoretical basis and candidate diagnostic indicators for tumors.

## 1 Introduction

Comparative genomic analyses have identified that at least 5% of the human genome is under purifying selection or evolutionary constraint, with ∼1.5% of the constrained sequences corresponding to orthologous coding genes. In contrast, the remaining approximately 3.5% are conserved elements in non-coding regions (Leypold and Speicher, 2021). In recent years, the evolutionary conservation of non-coding genomic regions has been discussed with multiple dimensions, such as spatiotemporal expressions and chromatin structure (Diehl et al., 2020; Gorkin et al., 2020). Nowadays, accumulated evidence has proven that long non-coding RNAs (lncRNAs) are involved in increasing numbers of biological processes (Yao et al., 2019). However, concerns remain regarding their conservation and their impact on corresponding functions, as well as on further phenotypic variability or disorders.

It is known that lncRNAs evolve more rapidly than mRNAs and that most lncRNAs currently need to be functionally uncharacterized or less annotated (Yao et al., 2019). Although lncRNAs have substantially fewer sequence conservation patterns than protein-coding genes across species, it is believed that evolutionarily conserved lncRNAs are likely to be functional and emerge as important targets to investigate conserved lncRNAs undergoing conserved processing, localization, and functions. Studies have revealed that some lncRNA orthologs exhibit different subcellular localizations in human and mouse embryonic stem cells due to differential RNA processing, which leads to their functional divergence in pluripotency regulation (Guo et al., 2020). Meanwhile, some extremely conserved non-coding regions are non-randomly distributed across chromosomes and tend to cluster in the vicinity of genes with regulatory roles in multicellular development and differentiation (Katzman et al., 2007; Polychronopoulos et al., 2017). In addition, many regulatory roles could be inferred by sequence conservations, such as the transcription factor (TF) recognition sites and the DNase I hypersensitive sites (DHSs) (Stergachis et al., 2014; Vierstra et al., 2014). Moreover, there are emerging diagnostic approaches working on transcription start sites, TF-binding sites, and other non-coding regions (Ulz et al., 2019). Ribo-seq studies have documented that there are more RNA–protein interaction signals among human-conserved and mouse-conserved lncRNAs than non-conserved lncRNAs, suggesting the significant roles for these conserved non-coding sequences (Ruiz-Orera and Alba, 2019). Moreover, a series of neuronal tissue-specific lncRNAs between the human and *Rhesus macaque* was found to possess conserved expression patterns during prefrontal cortex development and maturation (He et al., 2014). Definitely, studies argue that conserved non-coding molecular structures or functions are not necessarily dependent on sequence constraint to some extent (Diederichs, 2014; Leypold and Speicher, 2021). However, most of the comparative analyses based on sequence conservation still provided direct explanations for the alterations which are associated with phenotypes such as cancer (Rheinbay et al., 2020), malformations, behavioral and neurological disorders, and autism (Turner et al., 2017; Dickel et al., 2018). The understanding of the conservation of lncRNAs and their impacts on the underlying mechanisms, associated phenotypes, and diseases remains incomplete.

Here, on lncRNA sequences, we determined a highly conserved region (HCR) to verify their conservations and conducted homologous lncRNA clustering in humans and mice based on the detection of HCRs. Additionally, on the corresponding homolog clustering, we explored the potential function inference via HCRs on representative lncRNAs. For lncRNA *XACT*, we investigated the potential functional competence between *XACT* and lncRNA *XIST* by recruiting miRNA-29a, regulating the downstream target genes. Furthermore, for lncRNA *LINC00461*, we examined the interaction relationship between *LINC00461* and SND1, and the association may be perturbed during the progression of glioma. In addition, we constructed a website with user-friendly web interfaces for searching, analyzing, and downloading to present the homologous clusters of humans and mice.

## 2 Materials and methods

### 2.1 HCR definition, detection, and homolog clustering

In our study, we evaluated the conservation score of sequences using phastCons. Since the phyHMM algorithm built into phastCons relies on multiple sequence alignments and phylogenetic relationships among multiple sequences, our homologous sequence clustering was implemented through the following steps: 1) sequence search based on the Basic Local Alignment Search Tool (BLAST) (parameter: e-value < 1e-1) (Altschul et al., 1990); 2) multiple sequence alignment using MUSCLE (Edgar, 2004) and optimization of the alignment results based on trimAl (Capella-Gutiérrez et al., 2009) (parameter: -automated1); 3) building a phylogenetic tree based on the multiple sequence alignment results using FastTree (Price et al., 2010); 4) selecting a computational model using phyloFit based on the multiple sequence alignment results and phylogenetic analysis results (parameter: subst-mod HKY85); 5) obtaining the conservation score of the corresponding bases in the sequence using phastCons based on the multiple sequence alignment results and phylogenetic analysis results, as well as the model file generated by phyloFit (Siepel and Haussler, 2004); 6) identifying the HCRs based on the conservation scores along the sequence of each base. In our analysis, two criteria were set for identifying HCRs: (a) using a sliding window of 200 bp with a shift unit of 1 bp, we defined HCRs as contiguous segments with conservation scores of more than 60% sites> average conservation score of the whole sequence; (b) randomly selecting equal numbers of sequences from the HCR and non-HCR parts with a random length and satisfying the significance test of inter-group differences in conservation scores (*p* < 0.05). Furthermore, successful clustering of homologous lncRNAs in our study requires the capture of at least one HCR.

A total of 54,291 transcripts of human lncRNAs were obtained from the GENCODE version41 (http://www.gencodegenes.org/), and 25,419 transcripts of mouse lncRNAs were from GENCODE - Mouse Release M30 (gencodegenes.org), with the longest transcript of individual gene locus being collected. Homologous clustering was conducted individually in human lncRNAs, mouse lncRNAs, and their collection dataset.

### 2.2 Positionally conserved lncRNA detection

In our study, a region including a lncRNA and its nearest upstream two gene loci and downstream two gene loci were used to assess the collinearity between two positions. If the total of the upstream and the downstream four genes exhibited to be orthologs (with reciprocal best BLAST hits; RBH) and the lncRNA pair exhibited to be homologs by the BLAST, these lncRNAs were defined as positionally conserved lncRNA pairs.

### 2.3 Repeat detection, subcellular localization prediction, RNA–protein interaction, and RNA secondary structure prediction

Repeat detection on the sequences was carried out by RepeatMasker (Chen, 2004). The subcellular localization predictions were conducted by iLoc-LncRNA (lin-group.cn) (Su et al., 2018). RNA–protein interaction pairs were predicted by catRAPID (Bellucci et al., 2011). RNAfold was utilized to predict the secondary structure (http://rna.tbi.univie.ac.at/cgi-bin/RNAWebSuite/RNAfold.cgi) using the complete sequences, with the minimum free energy structure, the thermodynamic ensemble of the RNA structure, and the centroid structure, together with the positional entropy for each position. Among them, the centroid is the structure in the entire ensemble that has the minimum total base-pair distance to the structures in a given set of structures, which acts as an efficient method for predicting the RNA secondary structure. The high peaks in the plot indicate the more stable RNA structures.

### 2.4 miRNA-binding site scan and expression profiles

Potential binding sites were conducted by an online toolkit TargetScan (Agarwal et al., 2015) and miRanda (Enright et al., 2003). In addition, the expression of genes and lncRNAs was obtained and calculated from The Cancer Genome Atlas (TCGA; https://portal.gdc.cancer.gov; including 529 LGG samples and 173 GBM samples) and 1,152 normal brain cortex from the Genotype-Tissue Expression (GTEX) dataset (Consortium, 2013). The details are summarized in Supplementary Table S1. The batch correction was performed using the normalizeBetweenArrays function from the limma package (R package) (Ritchie et al., 2015; Chazarra-Gil et al., 2021).

### 2.5 Statistics

The statistical significance of differences between the two groups was analyzed by the paired Student’s *t-*test. All reported *p-*values were two-sided, and *p <* 0.05 was considered statistically significant.

### 2.6 Construction of the website

Our website was implemented based on Django (http://www.djangoproject.com). The web interfaces were developed by HTML5, CSS3, AJAX (Asynchronous JavaScript and XML), and in-house Python scripts.

## 3 Results

### 3.1 Homologous lncRNA clustering by sequence conservation

To investigate homologous relationships, we carried out homolog clustering based on sequence conservation. The conservation scores were calculated to identify homologous sequences. The clusters with successful detection of HCR were defined as lncRNA homologous clusters. We performed homolog clustering individually in human lncRNAs, mouse lncRNAs, and their collection dataset. The size distribution of all detected HCRs showed that the peak length of mouse HCRs was approximately 220 bp, whereas there were two peak lengths of human HCRs at approximately 210 and 410 bp, respectively (Supplementary Figure S1).

**FIGURE 1:**
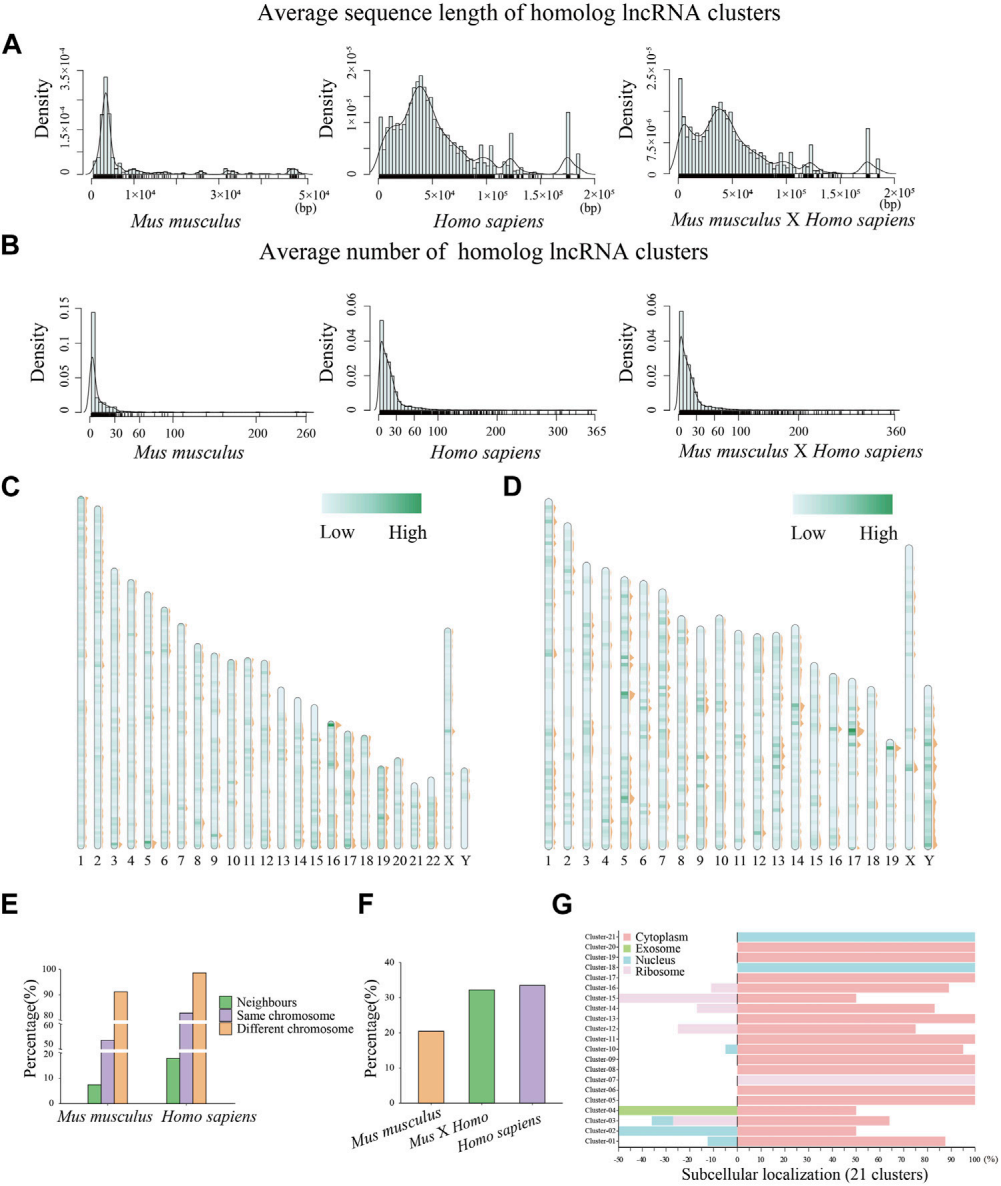
Distribution of divergent characteristics of human and mouse lncRNA clusters. **(A)** The average sequence length of homologous lncRNA clusters. **(B)** The average sequence number of homologous lncRNA clusters. **(C)** Scattered distributions of lncRNAs being clustered on human chromosomes. **(D)** Scattered distributions of lncRNAs being clustered on mouse chromosomes. **(E)** Percentage of the sequences in homologous clusters from different/same chromosomes and neighbor homologous lncRNAs (with an adjacent locus on the same strand) on the chromosome in humans and mice. **(F)** Percentage of sequences conservation clusters involving position conservation. **(G)** Subcellular localization patterns of sequence-conserved lncRNAs in individual clusters.

In our analysis, a total of 5,287 homologous clusters involving 6,166 lncRNAs were obtained in *Homo sapiens*, and 514 homologous clusters involving 1,610 lncRNAs were achieved in *Mus musculus*. Moreover, 21 clusters were detected containing both human and mouse lncRNAs. The limited numbers of lncRNAs across these two species consistently indicate that lncRNAs evolve fairly rapidly. The homologous lncRNAs of humans tend to possess longer sequences with an average length of 10^5^ bp (peak of ∼53,619 bp), with 10^4^ bp in the mouse (peak of ∼8,279 bp) (Figure 1A). Meanwhile, on average, there are more sequences being clustered into a homologous cluster in *H. sapiens* than in *M. musculus* (Figure 1B). Furthermore, we examined the distribution of homologous lncRNAs on chromosomes and found that the homologs were scattered and distributed throughout the chromosomes (Figures 1C, D). In order to investigate the potential impact of highly repeated regions on the identification of HCR on lncRNAs, we examined the distribution of mobile elements on lncRNAs and found that 18.31% of human HCRs (25.13% of mouse HCRs) possess the repeat elements. After the removal of labeled repeat sequences, the length distribution of human and mouse sequences, and that of HCRs was examined (Supplementary Figure S2). Accordingly, we choose to add a warning label to such HCR regions, informing us of the presence of repetitive sequence feature elements in that region. Additionally, the vast majority of the sequences in homologous clusters come from different chromosomes predictably, and there are 943 human homologous clusters (17.8%) that possess neighbor lncRNAs (with an adjacent locus on the same strand) on the chromosome, and 38 mouse clusters (7.4%) possess neighbor lncRNAs (Figure 1E). Based on this result, we further detected the genome position conservations of lncRNAs between the human and mouse (Method) and distributed the percentage of sequence conservation clusters involving position conservation (Figure 1F). Although lncRNAs are less conserved than coding genes, we assumed that homologous lncRNAs possibly possess functional conservation related to HCRs. Accordingly, based on our HCR detection method, we explored the homologous lncRNAs between the human and mouse and obtained 21 homologous clusters with each cluster including at least one mouse lncRNA and one human lncRNA. These clusters are summarized in Table 1. We could examine the specific functional roles that are similar or related since at least one HCR has been detected in each individual cluster. Meanwhile, among them, there are 11 homologous clusters containing only one lncRNA from the human and one from the mouse. Another 10 clusters contain more than two sequences which indicate the duplications in the corresponding species. Moreover, due to the remarkable sequence length, we could find that some clusters have overlapped lncRNA sequences. For instance, *snhg14* (with a length of 24,124 bp) and *MALAT* (with a length of 8,762 bp) both belong to two clusters, and *XACT* (with a length of 347,561 bp) belongs to seven clusters. As different clusters possess different HCRs, we chose to present the original clustering instead of integrating the overlapping clusters into a super cluster. Furthermore, in order to explore the spatial function characteristic of sequence-conserved lncRNAs, we retrieved sequence-conserved lncRNAs and compared their subcellular localization patterns (Figure 1G). The results show that most of the sequence-conserved lncRNAs in individual clusters possess similar subcellular localization patterns, inferring their conserved functional roles.

**TABLE 1:** Information on 21 homologous clusters between human and mouse lncRNAs.

Cluster	Chromosome	Gene ID	Gene name
Cluster1	Chr3	ENSG00000224424.8	*PRKAR2A-AS1*
	Chr7	ENSMUSG00000100826.7	*Snhg14*
	Chr8	ENSG00000261670.1	*
	Chr12	ENSG00000281344.1	*HELLPAR*
	Chr15	ENSG00000224078.15	*SNHG14*
	Chr15	ENSG00000261069.3	*
	Chr22	ENSG00000280434.1	*
	ChrX	ENSG00000241743.4	*XACT*
Cluster2	Chr11	ENSG00000255717.9	*SNHG1*
	Chr19	ENSMUSG00000108414.3	*Snhg1*
Cluster3	Chr10	ENSMUSG00000112117.4	*Rmst*
	Chr12	ENSG00000255794.11	*RMST*
Cluster4	Chr2	ENSMUSG00000102424.3	*Paupar*
	Chr11	ENSG00000281880.2	*PAUPAR*
	ChrX	ENSG00000241743.4	*XACT*
Cluster5	Chr9	ENSMUSG00000111841.2	*Gm20745*
	ChrX	ENSG00000241743.4	*XACT*
Cluster6	Chr6	ENSG00000272168.10	*CASC15*
	Chr13	ENSMUSG00000113216.3	*Gm40841*
Cluster7	Chr2	ENSMUSG00000052248.18	*Zeb2os*
	Chr2	ENSG00000238057.10	*ZEB2-AS1*
Cluster8	Chr5	ENSG00000245526.13	*LINC00461*
	Chr13	ENSMUSG00000050334.14	*C130071C03Rik*
Cluster9	Chr6	ENSMUSG00000086427.9	*Hoxa11os*
	Chr7	ENSG00000240990.10	*HOXA11-AS*
Cluster10	Chr5	ENSMUSG00000120166.1	*
	Chr5	ENSMUSG00000105044.2	*Gm10416*
	Chr5	ENSMUSG00000120227.1	*
	Chr6	ENSMUSG00000096299.5	*Gm21814*
	Chr7	ENSMUSG00000074887.6	*EU599041*
	Chr7	ENSMUSG00000120477.1	*
	Chr8	ENSMUSG00000110605.3	*Gm32856*
	Chr12	ENSMUSG00000113959.2	*Gm46339*
	Chr12	ENSMUSG00000116606.2	*Gm10479*
	Chr13	ENSMUSG00000110393.4	*Gm36445*
	Chr13	ENSMUSG00000112964.3	*E430024I08Rik*
	Chr13	ENSMUSG00000113019.3	*Gm47467*
	Chr13	ENSMUSG00000113047.3	*Gm47469*
	Chr13	ENSMUSG00000113204.3	*Gm46430*
	Chr17	ENSMUSG00000060149.9	*BC002059*
	Chr17	ENSMUSG00000120995.1	*Gm51425*
	Chr17	ENSMUSG00000116802.3	*Gm5165*
	Chr17	ENSMUSG00000072761.12	*Gm6712*
	Chr17	ENSMUSG00000066057.9	*Gm1976*
	Chr17	ENSMUSG00000095193.4	*Gm20939*
	Chr19	ENSG00000269349.1	*
Cluster11	Chr1	ENSG00000238063.3	*LINC01685*
	Chr4	ENSMUSG00000103541.2	*Gm37667*
Cluster12	Chr7	ENSMUSG00000100826.7	*Snhg14*
	Chr12	ENSG00000281344.1	*HELLPAR*
	Chr12	ENSG00000247373.3	*TMED2-DT*
	Chr15	ENSG00000224078.15	*SNHG14*
	Chr15	ENSG00000244879.10	*GABPB1-AS1*
	Chr22	ENSG00000279738.1	*
	Chr22	ENSG00000280434.1	*
	ChrX	ENSG00000241743.4	*XACT*
Cluster13	Chr2	ENSMUSG00000100303.5	*2600014E21Rik*
	Chr2	ENSG00000224577.4	*LINC01117*
Cluster14	Chr5	ENSG00000289731.1	*FAM153B*
	Chr6	ENSG00000203875.13	*SNHG5*
	Chr12	ENSG00000281344.1	*HELLPAR*
	Chr12	ENSMUSG00000021268.19	*Meg3*
	Chr14	ENSG00000214548.18	*MEG3*
	Chr15	ENSG00000244879.10	*GABPB1-AS1*
	Chr17	ENSG00000285877.1	*
	Chr17	ENSG00000279066.1	*HEXD-IT1*
	Chr19	ENSG00000267519.6	*MIR23AHG*
	Chr20	ENSG00000285796.1	*
	Chr22	ENSG00000280007.1	*
	Chr22	ENSG00000279217.1	*
	Chr22	ENSG00000279738.1	*
	Chr22	ENSG00000279080.1	*
	Chr22	ENSG00000280383.1	*
	ChrX	ENSG00000270641.1	*TSIX*
	ChrX	ENSG00000230590.13	*FTX*
	ChrX	ENSG00000241743.4	*XACT*
Cluster15	Chr2	ENSG00000223960.9	*CHROMR*
	Chr11	ENSG00000251562.10	*MALAT1*
	Chr11	ENSG00000289740.1	*TALAM1*
	Chr19	ENSMUSG00000092341.5	*MALAT1*
	Chr20	ENSG00000285796.1	*
	Chr22	ENSG00000278920.1	*
	Chr22	ENSG00000279217.1	*
	ChrX	ENSG00000241743.4	*XACT*
Cluster16	Chr3	ENSMUSG00000102652.2	*Gm37078*
	Chr5	ENSMUSG00000104793.2	*Gm43756*
	Chr6	ENSMUSG00000098318.9	*Lockd*
	Chr7	ENSMUSG00000098041.2	*Gm26981*
	Chr8	ENSMUSG00000110661.2	*Gm31805*
	Chr9	ENSMUSG00000097617.4	*Gm10687*
	Chr19	ENSMUSG00000092341.5	*MALAT1*
	Chr11	ENSG00000251562.10	*MALAT1*
	Chr11	ENSG00000289740.1	*TALAM1*
Cluster17	Chr12	ENSMUSG00000114826.3	*Gm10000*
	Chr14	ENSG00000285205.2	*
Cluster18	Chr2	ENSMUSG00000102869.4	*Norad*
	Chr12	ENSG00000257599.5	*OVCH1-AS1*
	Chr20	ENSG00000260032.2	*NORAD*
	ChrX	ENSG00000284618.1	*
Cluster19	Chr5	ENSG00000279726.2	*
	Chr18	ENSMUSG00000073594.4	*Gm10545*
Cluster20	Chr1	ENSMUSG00000053332.15	*Gas5*
	Chr1	ENSG00000234741.10	*GAS5*
	Chr15	ENSG00000244879.10	*GABPB1-AS1*
	Chr19	ENSG00000267519.6	*MIR23AHG*
	Chr20	ENSG00000285796.1	*
	Chr22	ENSG00000279159.1	*
	Chr22	ENSG00000278920.1	*
	Chr22	ENSG00000279738.1	*
	Chr22	ENSG00000279080.1	*
	ChrX	ENSG00000230590.13	*FTX*
	ChrX	ENSG00000241743.4	*XACT*
Cluster21	Chr6	ENSMUSG00000104222.2	*Gm7292*
	Chr12	ENSG00000257599.5	*OVCH1-AS1*
	Chr20	ENSG00000260032.2	*NORAD*
	ChrX	ENSG00000284618.1	*

Note: “*” indicates that the sequences are not found in the RefSeq database.

### 3.2 Functional exploration of homologous lncRNAs between the human and mouse

Our study aims to investigate the potential functional conservation of homologous lncRNAs by detecting HCRs. As a case study, we focused on *XACT*, a known X-linked lncRNA that has been reported to coat active X chromosomes in early human embryonic stages (Vallot et al., 2013). Previous studies have shown that *XACT* is weakly conserved across mammals and absent in mice, suggesting that it may have evolved to fulfill a primate-specific function (Vallot et al., 2013). To explore the potential functional conservation of *XACT*, we examined its sequence conservation and found that it belongs to seven homologous clusters. This suggests that *XACT* may have conserved functional roles in both humans and mice despite its weak sequence conservation. Our findings suggest that homologous lncRNAs detected by HCR analysis may have functional conservation and could provide a basis for further investigation of lncRNA functions.

**FIGURE 2:**
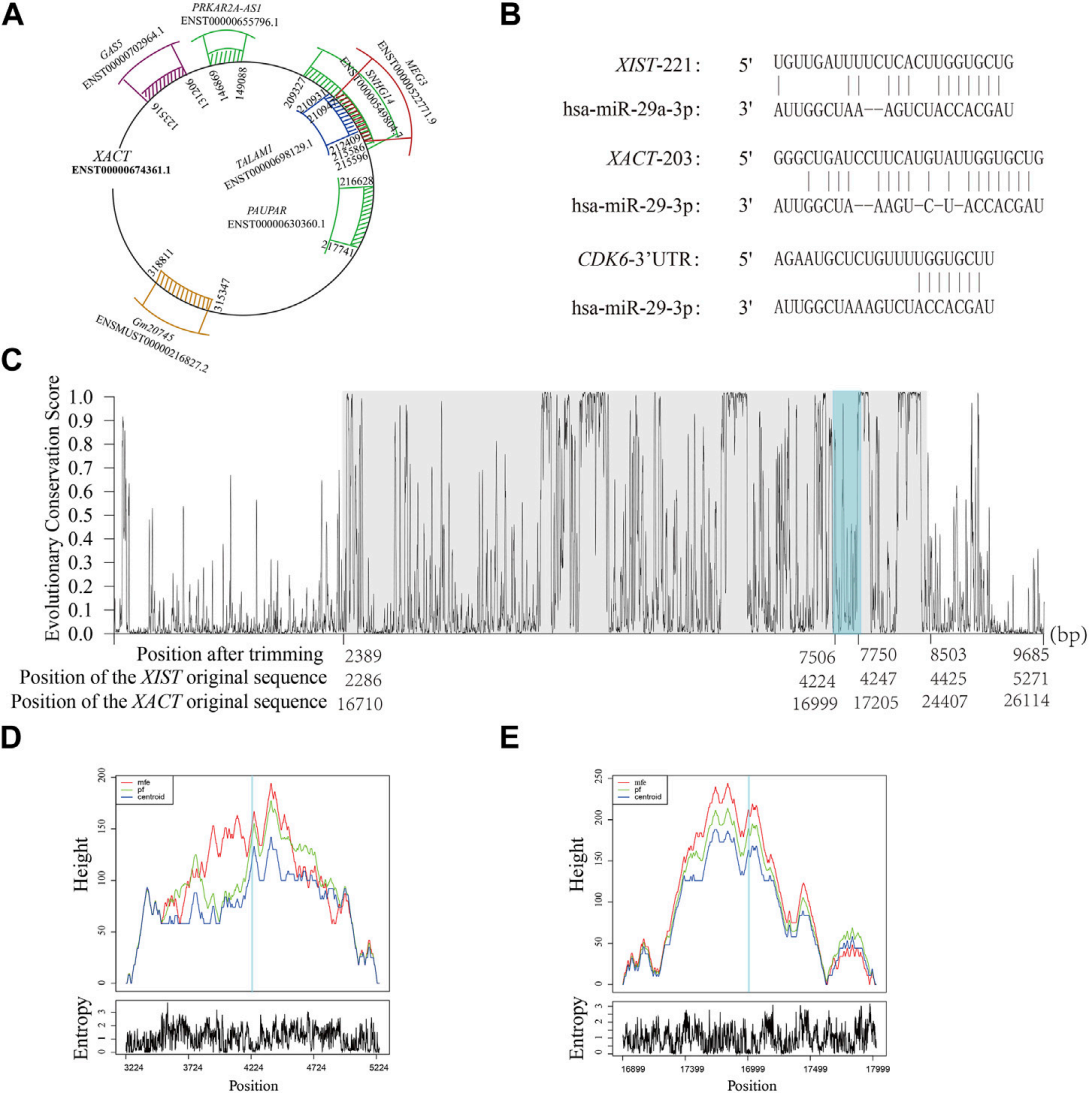
Sequence exploration on *XACT*. **(A)** Different HCRs on *XACT*, with the representative sequence IDs from each homologous cluster. **(B)** Binding sites of miR-29a with *XIST* and *XACT*. **(C)** Detection of HCRs (with a gray background) on the transcripts of *XIST* and *XACT* and the binding region of miRNA-29a (with a steel blue background). **(D, E)** Mountain plots for **(D)** *XIST* and **(E)** *XACT* of the MFE (minimum free energy) structure, the thermodynamic ensemble of the RNA structure, and the centroid structure, together with the positional entropy for each position.

In our analysis, we found that *XACT* has diverse HCRs with different transcripts of lncRNAs, including *GAS5* (ENST00000702964.1; HCR location: 361∼767 bp), *PRKAR2A-AS1* (ENST00000655796.1; HCR location:146,869∼149,088 bp), *SNHG14* (ENST00000549804.7; HCR location: 8,498∼14,987 bp), *TALAM1* (ENST00000698129.1; HCR location: 136∼2,060 bp), *MEG3* (ENST00000522771.9; HCR location: 1,493∼8,653 bp), *PAUPAR* (ENST00000630360.1; HCR location: 48∼1,234 bp), and *Gm20745* (ENSMUST00000216827.2; HCR location: 8∼3,778 bp), inferring its complicated roles suggesting that *XACT* may have related or supplementary functions with sequences in the same homologous clusters. Our analysis revealed that *XACT* belongs to seven clusters, each having its own HCR information and seven different mouse lncRNAs (Figure 2). However, we did not find any mouse lncRNA that belonged to seven or fewer different clusters like *XACT*. As a result, we could not detect the ortholog of *XACT* in mice consistently. In other words, *XACT* appears to be a primate-specific lncRNA that has weak conservation across mammals, as suggested by previous studies. Recently, a study reported that *XACT* and lncRNA *XIST* compete in controlling X chromosome activity during early human development, but the underlying mechanism remains unclear (Vallot et al., 2017). Previous research has shown that *XIST* regulates gene expression by recruiting miRNAs. Thus, we hypothesized that the regulatory roles of *XACT* and *XIST* may involve miRNAs. To investigate this, we examined a common HCR and found that miRNA-29a can bind to *XIST* at 4,424∼4,247 bp (Figure 2B). Additionally, predictive analysis suggests that miRNA-29a can also bind to *XACT* at 16,999 to 17,205 bp, indicating that both *XIST* and *XACT* may recruit miRNA-29a to regulate gene expression. Furthermore, we found that the binding region locates within the HCR of *XACT* and *XIST* (Figure 2C). For these two sequences, we presented the mountain plots of the MFE (minimum free energy) structure, the thermodynamic ensemble of the RNA structure, and the centroid structure, together with the positional entropy for each position (Figures 2D, E), and the binding regions exhibit to possess the most stable RNA structure. In addition, we found that *CDK6,* a kind of cyclin-dependent kinase, is the potential target gene of miRNA-29a, and *CDK6* is a dominant gene playing a significant role in cell proliferation (Goel et al., 2022). Evidence from the relative expression profiles of human early development shows that the major burst of zygotic *XIST* and *XACT* expression occurred at the four-cell and the eight-cell stages (Vallot et al., 2017), inferring the potential associations between *XIST*/*XACT* and *CDK6*, through recruiting miR-29a.

**FIGURE 3:**
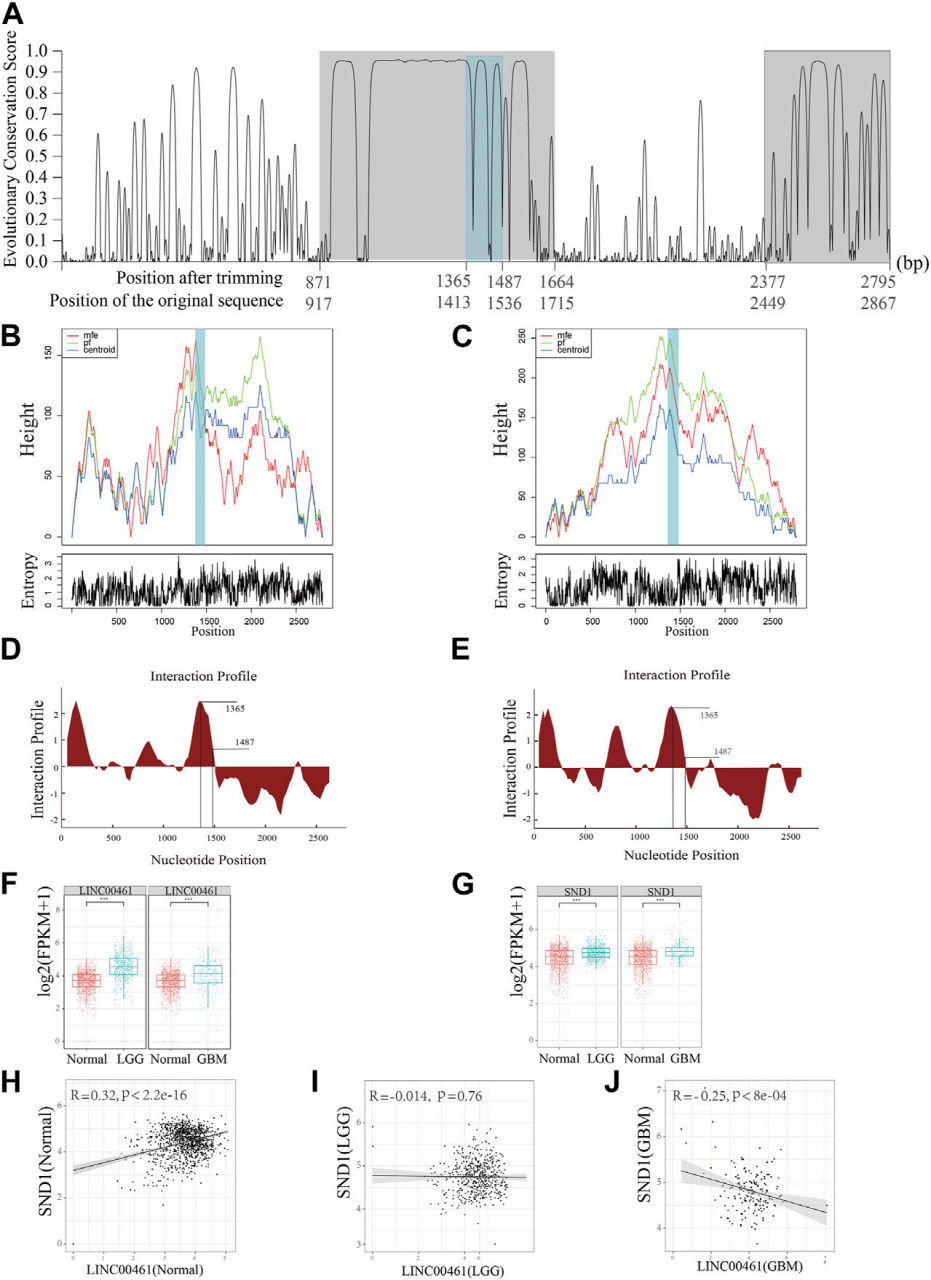
Functional prediction on the lncRNA *LINC00461*. **(A)** Detection of HCRs (with a gray background) on the transcript of *LINC00461* and the binding regions of SND1 (with a steel blue background). **(B, C)** Mountain plots of the MFE (minimum free energy) structure, the thermodynamic ensemble of the RNA structure, and the centroid structure, together with the positional entropy for each position. **(D)** Distribution of the binding ability of SND1 to *LINC00461* and **(E)** *C130071C03Rik* on an individual HCR region. **(F)** Comparison of the expression level of *LINC00461* in samples of normal and two different types of gliomas (LGG and GBM). **(G)** Comparison of the expression level of SND1 in samples of normal and two different types of gliomas. **(H**–**J)** Correlation between expressions of SND1 and *LINC00461* in **(H)** normal samples, **(I)** LGG samples, and **(J)** GBM samples. *** indicates *p* < 10^−4^.

Although the structure–function relationship is well established for many proteins, the same cannot be said for most lncRNAs as they are still largely uncharacterized. Moreover, due to the lack of understanding of their functions, it is challenging to identify lncRNA biomarkers that are involved in cancer development. Based on the detection of HCRs of the homologous clusters, ENSMUST00000242216.1 (from mouse; *C130071C03Rik*) and ENST00000658935.1 (from human; ECONEXIN; *LINC00461*) are found to be in a homologous cluster. By comparing their subcellular localization results, we discovered that these two homologous lncRNAs both perform their functions in the cytoplasm. Two HCRs were found along the *LINC00461* sequence (917∼1,715 bp and 2,449∼2,867 bp on ENST00000658935.1; Figure 3A; the region with a gray background color). Moreover, based on the examination of the binding ability of the lncRNAs, we found that SND1 was able to bind to *LINC00461* and *C130071C03Rik* on the HCR region (Figure 3A, the region with a steel blue background) with highly reliable values (interaction propensity:96.08; z-score:3.6; Figures 3D, E), indicating the significantly associated roles of SND1 and these two lncRNAs. Furthermore, we hypothesized that, given the possibility of interactions, the binding region on the HCR should possess a stable spatial structure. To emphasize the key findings, we presented three types of structures (the minimum free energy, the thermodynamic ensemble, and the centroid structure) for the RNA molecule, along with the positional entropy data for each position (Figures 3B, C). By analyzing the data, we observed that the binding region (∼1,500 bp) exhibited the most stable RNA structure, which aligns with its presumed functional significance. Therefore, our results suggest a correlation between the stability of the RNA structure and its potential biological function in this specific context.

Due to this fact, glioma is divided into lower-grade glioma (LGG) and high-grade glioma (e.g., glioblastoma, GBM), and *LINC00461* is previously reported to be a regulator in glioblastoma (GBM) (Deguchi et al., 2017). In our analysis, we further detected the expression patterns of *LINC00461* and SND1 among different types of gliomas and the normal brain cortex using the TCGA and GTEX datasets. The expressions of *LINC00461* in GBM and LGG are significantly higher than those in normal samples (Figure 3F); the expressions of SND1 in GBM and LGG are also significantly higher than those in normal samples (Figure 3G), suggesting our predicted potential associated regulator roles of *LINC00461* and SND1. Furthermore, considering the interaction possibility between *LINC00461* and SND1, we explored the Pearson correlation relationships between expressions of these two sequences among different sample types. Intriguingly, we found that in normal samples, the expressions of SND1 and *LINC00461* are positively correlated (Figure 3H; *R* = 0.32, *p* < 2.2e-16), whereas the correlation relationship is negatively correlated but exhibited to be not significant in LGG samples (Figure 3I; *R* = −0.014, *p* = 0.76). Meanwhile, the negative correlation relationship is enhanced in GBM samples (Figure 3J; *R* = −0.25, *p* < 8e-4). Collectively, our results indicate the interaction relationship between *LINC00461* and SND1, and their interaction or association may be perturbed during the progression of glioma, which provides a candidate potential marker for glioma.

### 3.3 Website for homologous cluster searching

**FIGURE 4:**
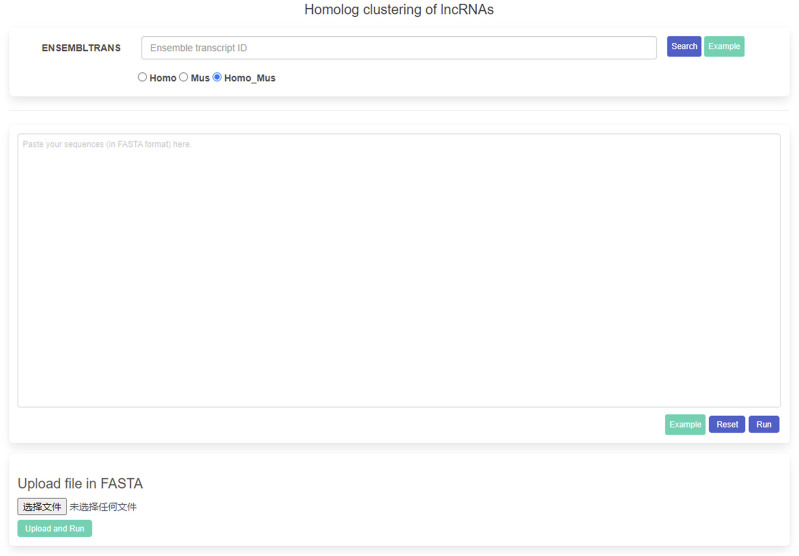
Snapshot of the homepage of the website on homolog clustering.

In order to present the homologous clusters of the human and mouse, we constructed a website with user-friendly web interfaces for searching, analyzing, and downloading (Figure 4). The website was implemented based on Django (http://www.djangoproject.com). The web interfaces were developed by HTML5, CSS3, AJAX (Asynchronous JavaScript and XML), and in-house Python scripts. It is freely available online at http://homolog.cn/. A user could search the homologous clusters and the detected HCR result by a lncRNA ID or by lncRNA sequences directly, and the website would return the related homologous cluster results, including the BLAST results, the phylogenetic trees among the homologs, the conservation scores along the sequences, and the HCR detection file. We will also provide clear instructions with a README file in the output file folders on how to interpret the results. In addition, a warning label to HCRs, informing users of the presence of repetitive sequence feature elements will be returned and displayed in the HCR results during sequence search. However, our website nowadays can only identify the lncRNA IDs from Ensembl transcripts. Furthermore, we have added the example inputs and outputs to guide the user. It is recommended that a user could paste the lncRNA sequences in the textbox or upload the lncRNA sequences by the “upload” button. If our website receives the uploaded sequences, the BLAST will be invoked and the related homologous cluster results of its best-hit lncRNA will be returned. We think the website will provide direct access for a biologist who is interested in sequence conservation for their corresponding functional inference in humans and mice.

## 4 Discussion

### 4.1 Functional lncRNA exploration

Most of the conservations are just based on lncRNA sequence alignments by BLAST. We believe that the detection of highly conserved segments, such as HCR in our analysis, acts as an improvement of the conservation annotation, and the functional conservation could be inferred more relevantly. Among the 21 homologous clusters we identified, some lncRNAs were detected to be highly conserved between humans and mice (Table 1), such as *MALAT*, *snhg1*, *Paupar*, and *Zeb2*. The successful detection of these extremely conserved regions in these clusters would provide functional references. In our study, we only focused on *XACT*, which acts as an overlapped lncRNA in different clusters, due to its remarkable length, and discussed its possible functional roles by recruiting miRNA-29a on the HCR. *XIST*, which shares a common HCR with *XACT*, is reported to possess the binding sites of miRNA-29a on the HCR region, and the regulation of *XIST* and miR-29a was reported in the denatured dermis and human skin fibroblasts (HSFs) after thermal injury (Guo et al., 2018) but with limited documents on XCI. Taken together, our study provides a potential explanation of the competence of *XACT* and *XIST* in XCI. In addition, it is widely believed that lncRNA is closely associated with various diseases (Wang and Chang, 2011; Batista and Chang, 2013). In recent years, lines of evidence have accumulated that lncRNAs are involved in tumorigenesis and tumor metastasis, especially in cancer development (Taniue and Akimitsu, 2021), cancer immunity, cancer metabolism, and cancer metastasis (Jiang et al., 2019). Recent studies have shown that lncRNAs may also engage in remodeling the tumor microenvironment (Sang et al., 2018; Botti et al., 2019). Given the fact that cancer is difficult to cure, developing effective therapeutic approaches or markers to treat cancer is still important. Among the different types of cancers, gliomas are primary brain tumors derived from neuroglial stem or progenitor cells. Our study based on the detection of HCR and the functional exploration of this region offers a possibility that *LINC00461* and SND1 could act as candidate markers in different types of gliomas. The correlations are obviously changed in LGG and GBM. We hypothesized that this could be a result of the binding ability between *LINC00461* and SND1 during the progression of glioma. This finding could, to some extent, improve the diagnostics and classification systems. Certainly, our future experiment verification would be conducted in this field, and some substantial progress would be made based on this analysis in understanding the molecular pathogenesis of gliomas.

### 4.2 Future development of the website

The data on lncRNAs serve as an important resource for public databases, and the number of lncRNAs is increasing. Many important databases were constructed for providing the sequence information and related aspects of functional characteristics of lncRNAs using omics datasets, such that LNCipedia (Volders et al., 2015) includes human lncRNA transcript sequences and their structure information; lncRNADisease (Bao et al., 2019) distributes the associations between lncRNAs and diseases; LncRNA2Target (Cheng et al., 2019) collects and provides with RNA-seq datasets before and after knockdown or overexpression of some specific lncRNAs; LncRNA2Function (Jiang et al., 2015) correlates lncRNAs with Gene Ontology (GO) terms and biological process; and lncRNASNP2 (Miao et al., 2018) relates lncRNAs with SNPs and TF2LncRNA (Jiang et al., 2014) with transcription factors similarly. Our website here is not an integrated database, just providing the homologous cluster search results. Considering that limited databases focus on sequence conservations and that our detection of highly conserved segments could act as an improvement on the conservation annotation, the highly conserved information will enhance our understanding of functional lncRNAs. Meanwhile, we admit that nowadays, the species are finite in humans and mice, and the related result is limited. As more lncRNAs are identified in different species, we will continue to integrate more lncRNAs from other species, provide more characteristics of lncRNAs, such as lncRNA–DNA/RNA interactions, and modification regulations, editing site information in these specially conserved regions, and perform comparisons of lncRNAs in a relatively large evolutionary timescale.

## Data Availability

Publicly available datasets were analyzed in this study. Information for existing publicly accessible datasets is contained within the article.

## Supplementary material

SUPPLEMENTARY FIGURE S1 The length distribution of detected HCRs in human lncRNAs, mouse lncRNAs, and the collected dataset.

SUPPLEMENTARY FIGURE S2 The length distribution of **(A)** human and mouse sequences and **(B)** the HCRs after the flagged repeats are removed.
