# Supplementary figures and images for: Functional inference of long non-coding RNAs through exploration of highly conserved regions
Source: Front Genet. 2023 May 16;14:1177259. doi: 10.3389/fgene.2023.1177259 (PMC10229068; doi:10.3389/fgene.2023.1177259)

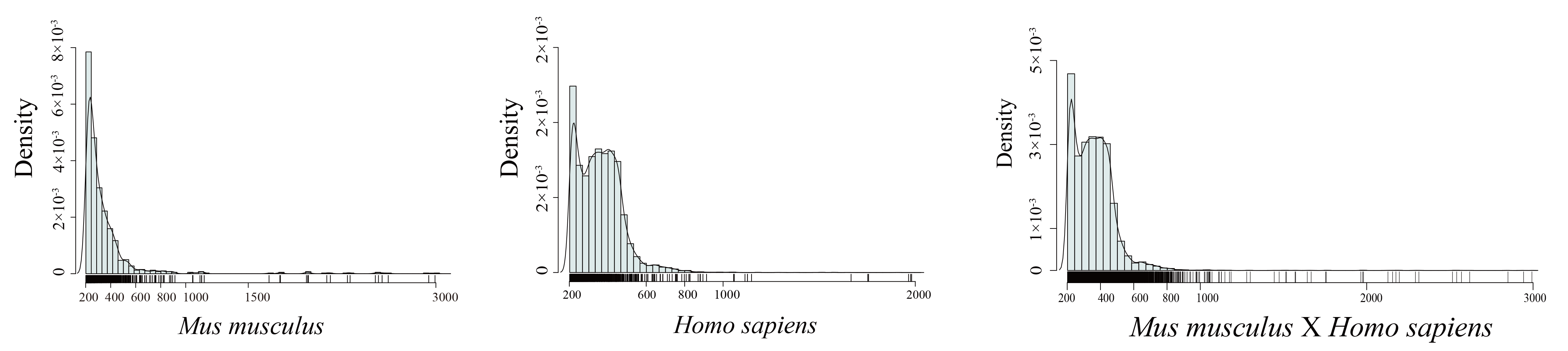

Supplement: Supplementary file 1 [file Image1.JPEG]

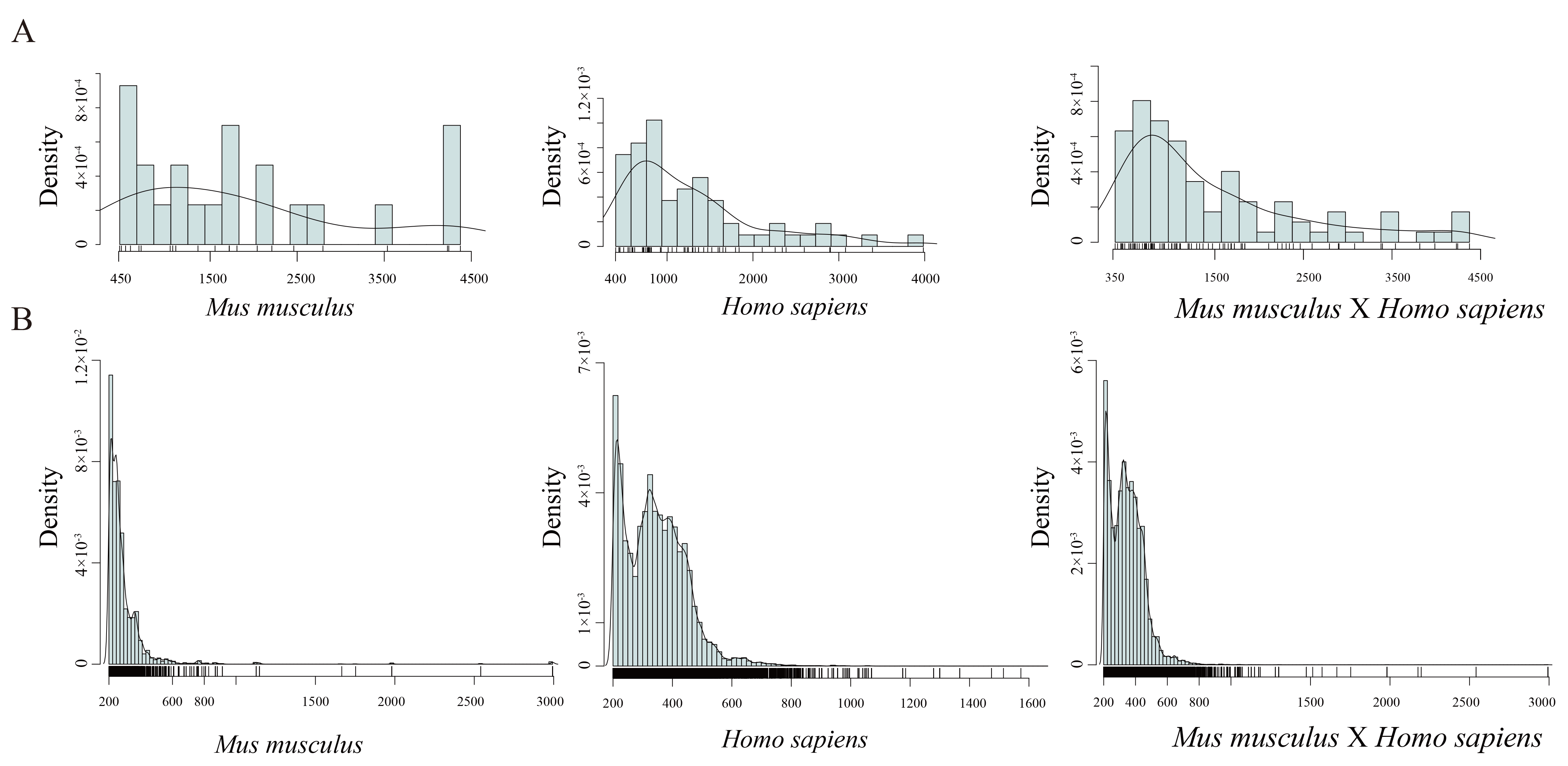

Supplement: Supplementary file 2 [file Image2.JPEG]
